# Novel Functions of Death-Associated Protein Kinases through Mitogen-Activated Protein Kinase-Related Signals

**DOI:** 10.3390/ijms19103031

**Published:** 2018-10-04

**Authors:** Mohamed Elbadawy, Tatsuya Usui, Hideyuki Yamawaki, Kazuaki Sasaki

**Affiliations:** 1 Laboratory of Veterinary Pharmacology, Department of Veterinary Medicine, Faculty of Agriculture, Tokyo University of Agriculture and Technology, 3-5-8 Saiwai-cho, Fuchu, Tokyo 183-8509, Japan; mohamed.elbadawy@fvtm.bu.edu.eg (M.E.); skazuaki@cc.tuat.ac.jp (K.S.); 2 Department of Pharmacology, Faculty of Veterinary Medicine, Benha University, Moshtohor, Elqaliobiya, Toukh 13736, Egypt; 3 Laboratory of Veterinary Pharmacology, School of Veterinary Medicine, Kitasato University, Towada, Aomori 034-8628, Japan; yamawaki@vmas.kitasato-u.ac.jp

**Keywords:** MAPK, DAPK, ERK, p38, JNK

## Abstract

Death associated protein kinase (DAPK) is a calcium/calmodulin-regulated serine/threonine kinase; its main function is to regulate cell death. DAPK family proteins consist of DAPK1, DAPK2, DAPK3, DAPK-related apoptosis-inducing protein kinases (DRAK)-1 and DRAK-2. In this review, we discuss the roles and regulatory mechanisms of DAPK family members and their relevance to diseases. Furthermore, a special focus is given to several reports describing cross-talks between DAPKs and mitogen-activated protein kinases (MAPK) family members in various pathologies. We also discuss small molecule inhibitors of DAPKs and their potential as therapeutic targets against human diseases.

## 1. Introduction: DAPKs, MAPKs

**Figure 1:**
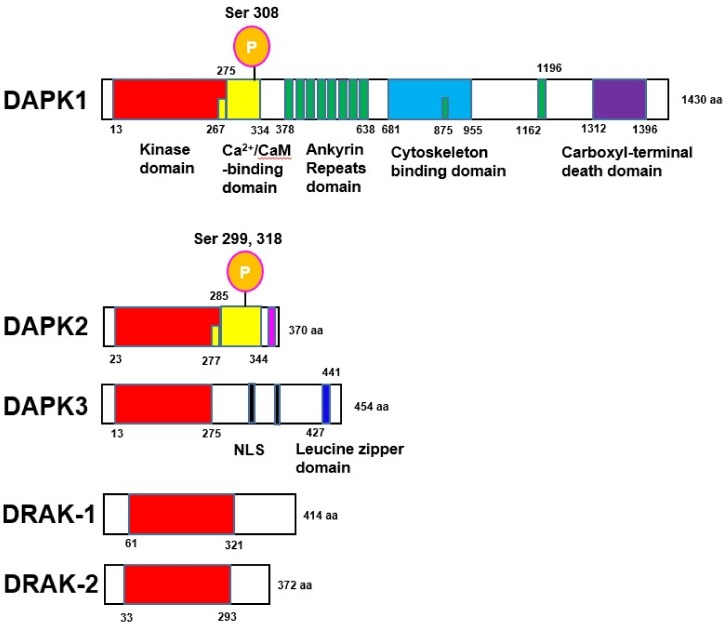
Structures of death associated protein kinase (DAPK) family proteins. DAPK1 possesses an N-terminal kinase domain, a Ca^2+^/Calmodulin (CaM)-binding domain, an ankyrin repeats domain, a cytoskeleton binding domain, and a carboxyl-terminal death domain. DAPK1 is auto-phosphorylated at Ser308, which blocks its CaM binding site and keeps the site inactivated. DAPK2 contains an N-terminal kinase domain, a Ca^2+^/CaM-binding domain, and a C-terminal tail with no homology to any known proteins. DAPK2 at Ser318 is dephosphorylated and promotes CaM binding to the autoregulatory domain. Phosphorylation of DAPK2 at Ser299 increases DAPK2 activity independently of CaM binding. DAPK3 has an N-terminal kinase domain, a leucine zipper domain, and two nuclear localization sequences (NLS). DRAK-1 and DRAK-2 have an N-terminal kinase domain. The location and total number of amino acids (aa) are shown. The recent domain information on human DAPK family proteins was collected from http://www.uniprot.org.

Death-associated protein kinase (DAPK) family proteins are closely related, Ca^2+^/calmodulin (CaM)-regulated serine/threonine kinases, whose members not only possess significant homology in their catalytic domains but also share cell death-associated functions [1,2]. DAPK family proteins include DAPK1, DAPK2, DAPK3, and DAPK-related apoptosis-inducing protein kinases (DRAK-1 and DRAK-2) [3,4,5,6,7] (Figure 1). DAPK1 has multiple complex domains including an N-terminal kinase domain, a Ca^2+^/CaM-binding domain, a series of ankyrin repeats, a cytoskeleton binding domain, and a carboxyl-terminal death domain. DAPK2 contains an N-terminal kinase domain with high homology to DAPK1 catalytic domain [5], a conserved CaM-binding autoregulatory domain, and a C-terminal tail with no homology to any known proteins [6]. DAPK3 has an N-terminal kinase domain, a leucine zipper domain, and two putative nuclear localization sequences (NLS) [8]. The kinase domain and death domain are both critical for its pro-apoptotic activity [1,2,9]. All of these kinases are closely related to each other, sharing about 80% identity in their kinase domains [3,6], except for DRAK-1 and DRAK-2, whose kinase domains are only 50% identical to DAPK1 [10]. 

Mitogen-activated protein kinases (MAPKs) are an important sub-family of non-receptor serine-threonine kinases. MAPKs mediate signal transduction pathways that are involved in cellular responses to a diverse range of stimuli, such as mitogens, hormones, osmotic stress, heat shock, proinflammatory cytokines, and significant developmental changes in organisms. They mediate cellular functions including proliferation, differentiation, mitosis, gene expression, and apoptosis [11]. Extracellular stimuli such as growth factors result in a sequential phosphorylation cascade that ultimately leads to activation of MAPKs. Once activated, MAPKs activate downstream signals and transcription factors. MAPKs mainly consist of the extracellular signal-regulated kinases (ERK1–8), p38 MAPKs (p38α–δ), and c-Jun N-terminal kinases (JNK1–3) [12]. While the ERKs are mainly activated in response to proliferative signals, p38s and JNKs are activated in response to various stresses. Although there are several MAPK isoforms, the best-investigated ones are ERK1/2, JNK1/2, and p38α.

The mutual regulation between MAPK and DAPK family proteins plays a role in apoptosis regulation and several diseases. In this review, we introduce the reports showing the regulatory mechanisms and various functions of DAPK family proteins. Furthermore, we refer to reports indicating the relationship between DAPK and MAPK family proteins in multiple diseases.

## 2. Cellular Functions of DAPK Family Proteins

Increased activity of DAPK family proteins results in pronounced death-associated cellular changes, which include cell rounding, membrane blebbing, detachment from extracellular matrix, and formation of autophagic vesicles [1,2,4,5,6,9,13,14,15,16,17,18,19].

Among DAPK family proteins, DAPK1 controls cell cycle, apoptosis, autophagy, tumor metastasis, and oxidative stress. Several reports demonstrate that complex regulation of DAPK1 activity by various signaling pathways modulates the balance between pro-apoptotic and pro-survival pathways [20]. Furthermore, DAPK1 has been implicated in autophagy induction upon endoplasmic reticulum (ER) stress [21].

DAPK2 is known to be involved in pro-inflammatory responses mediated by granulocytes, which might be linked to the mechanism of myosin light chain (MLC) phosphorylation by DAPK2 [22]. Besides, DAPK2 has also been associated with differentiation processes in the erythropoietic lineage. DAPK2 knock-in mice showed a decreased response to erythropoietin treatment, suggesting that DAPK2 might exert fundamental regulatory effects on pro-erythroblast development [23].

The biological role of DAPK3 has been gradually investigated [24]. DAPK3 is pro-apoptotic [3] and executes this function either by inducing apoptosis or activating autophagy with or without the involvement of caspase proteins [25,26]. DAPK3 was also shown to mediate inflammatory signals including L13a (ribosome protein), ERK, and interferon (IFN)-γ-activated inhibition of translation [27].

## 3. Regulation of DAPK Family Proteins

DAPK1, DAPK2, and DAPK3 are all ubiquitously expressed in various tissues, such as heart, lung, spleen, and brain. In particular, DAPK1 is highly expressed in the hippocampus [1,3,4,5]. 

DAPK1 acts as a positive mediator of apoptosis induced by several death stimuli, such as interferon (IFN)-γ, Fas, transforming growth factor (TGF)-β, tumor necrosis factor (TNF)-α, ceramide, oncogene expression, and DNA damaging agents [2,9,13,14,16,28,29,30]. In normal conditions, DAPK1 is auto-phosphorylated at Ser308, which blocks its CaM binding site and keeps it inactivated [31] (Figure 1). Once stimulated, DAPK1 is dephosphorylated and promotes CaM binding to the site, which induces apoptotic responses [32,33,34]. In these papers, it was also suggested that DAPK1 dephosphorylation is caused by a class III phosphoinositide (PI)3-kinase-dependent phosphatase.

DAPK2 is regarded as a tumor suppressor in non-solid tumors and is implicated in apoptotic cell death [22]. After stimulation, DAPK2 at Ser318 is dephosphorylated and promotes CaM binding to the autoregulatory domain. These processes release the inhibitory binding domain to DAPK2, which leads to the access of the substrate of DAPK2. Dephosphorylation of DAPK2 also enhances homodimerization of DAPK2, which promotes membrane blebbing [35]. Recently, DAPK2 was shown to be phosphorylated at Ser299 by cyclic guanosine monophosphate (cGMP)-dependent protein kinase 1 [36]. Phosphorylation of DAPK2 at Ser299 increases DAPK2 activity independently of CaM binding (Figure 1).

On the contrary, DAPK3 lacks a Ca^2+^/CaM regulatory domain, and its activity is regulated independently of intracellular Ca^2+^ levels. There are reports showing that DAPK3 is activated via interacting with DAPK1, forming a death-associated multi-protein complex [1,21]. DAPK3 was also shown to regulate DAPK1-induced apoptosis in HEK293T cells [37], and to promote starvation-induced autophagy through the regulation of Atg9-mediated autophagosome formation [38]. DAPK3 is activated in response to stress signals, and the cytosolic localization of DAPK3 may be a critical determinant in its pro-apoptotic activity [3,4,37]. The cytoplasmic distribution of DAPK3 may be regulated through its phosphorylation by DAPK1 [37]. This regulation of DAPK3 by DAPK1 suggests a mechanism by which a death signal can be transferred from one kinase to another in a catalytic amplification loop. Moreover, DAPK2 is capable of phosphorylating DAPK1 and DAPK3 [37], which is associated with cytoskeletal remodeling [1].

These studies suggest that the activity of DAPK proteins is regulated not only by the upstream signals but also the interactions by themselves. 

## 4. Cellular Functions of DAPK Family Proteins

Increased activity of DAPK family proteins results in pronounced death-associated cellular changes, which include cell rounding, membrane blebbing, detachment from the extracellular matrix, and formation of autophagic vesicles [1,2,4,5,6,9,13,14,15,16,17,18,19].

Among DAPK family proteins, DAPK1 controls cell cycle, apoptosis, autophagy, tumor metastasis, and oxidative stress. Several reports demonstrate that complex regulation of DAPK1 activity by various signaling pathways modulates the balance between pro-apoptotic and pro-survival pathways [20]. Furthermore, DAPK1 has been implicated in autophagy induction upon endoplasmic reticulum (ER) stress [21].

DAPK2 is known to be involved in pro-inflammatory responses mediated by granulocytes, which might be linked to the mechanism of myosin light chain (MLC) phosphorylation by DAPK2 [22]. Besides, DAPK2 has also been associated with differentiation processes in the erythropoietic lineage. DAPK2 knock-in mice showed a decreased response to erythropoietin treatment, suggesting that DAPK2 might exert fundamental regulatory effects on pro-erythroblast development [23].

The biological role of DAPK3 has been gradually investigated [24]. DAPK3 is pro-apoptotic [3] and executes this function either by inducing apoptosis or activating autophagy with or without the involvement of caspase proteins [25,26]. DAPK3 was also shown to mediate inflammatory signals including L13a (ribosome protein), ERK, and IFN-γ-activated inhibition of translation [27].

## 5. DAPKs in Disease

**Figure 2:**
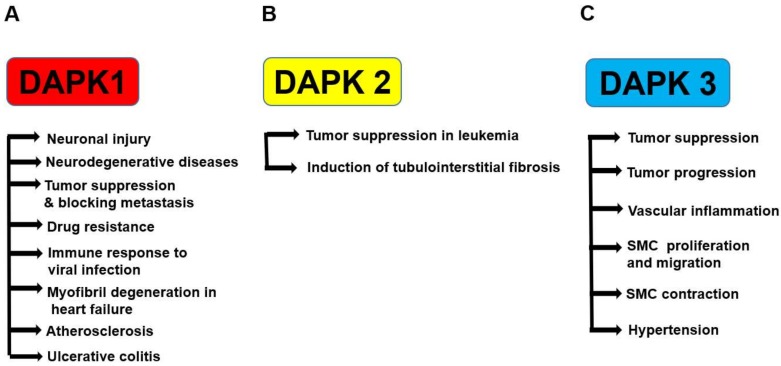
Roles of DAPK family proteins in various diseases. DAPK1 regulates neuronal injury, neurodegenerative diseases, tumor suppression, metastasis blocking, resistance to anti-cancer drugs, immune response during antiviral infection, myofibril degeneration in heart failure, atherosclerosis, and ulcerative colitis (**A**). DAPK2 regulates tumor suppression in leukemia and induction of tubulointerstitial fibrosis (**B**). DAPK3 regulates tumor suppression or progression, vascular inflammation, smooth muscle cell (SMC) proliferation and migration, SMC contraction, and hypertension (**C**).

Among DAPK proteins, the relationship between DAPK1 and diseases has been often reported (Figure 2A). In the developing and adult brain, DAPK1 is widely expressed [39,40]. In addition, elevated DAPK1 activity is detected following brain injury due to ischemia [41,42], seizure [43,44], epilepsy [45], and Alzheimer’s disease (AD) [46,47] as well as in response to ceramide and glutamate toxicity [28,48], indicating a close relationship between DAPK1 and neuronal cell death [49].

Two main characteristics of AD are amyloid-beta (Aβ) senile plaques and tau neurofibrillary tangles. The metabolism of amyloid precursor protein (APP) results in extracellular deposition of Aβ protein that leads to the formation of Aβ senile plaques. In AD, tau is commonly hyperphosphorylated prior to tangle formation and neurodegeneration. [50,51]. However, how tau accumulation and phosphorylation are deregulated in AD is not fully understood.

Several studies showed that DAPK1 contributes to the pathogenesis of AD through excessive processing of APP and [46] triggering hyperphosphorylation of tau. It was also shown that DAPK1 inhibits microtubule assembly and stability through activation of microtubule-affinity regulating kinase (MARK)1 and MARK2, which leads to phosphorylation of tau at Ser262 [52]. It was also demonstrated that DAPK1 expression is highly upregulated in human AD hippocampus tissues. In the report, DAPK1 knock-out mice exhibited a decrease in expression and stability of tau protein [53]. 

In addition to regulating APP and tau proteins, DAPK1 mediates the neuronal cell death in AD model animals. DAPK1 binds N-myc downstream regulated gene 2 (NDRG2), which triggers its phosphorylation at Ser350 and induces neuronal cell death in AD model mice. In contrast, DAPK1 inhibition prevented NDRG2-mediated neuronal cell death [54]. These data suggest that DAPK1 might be a novel therapeutic target for the treatment of human AD [53].

In a wide variety of tumors, DAPK1 expression is frequently suppressed and the tumor-suppressive function of DAPK1 is linked to its role in cell death via apoptosis and autophagy [20]. It was reported that DAPK1 expression was lost in tumors due to hypermethylation of the DAPK1 gene [55]. Moreover, it was shown that DAPK1 was capable of suppressing oncogenic transformation caused by c-Myc and E2F, which blocks the tumor metastasis [13,14]. DAPK1 is also mediated in anti-cancer drug resistance to 5-fluorouracil in endometrial adenocarcinoma cells [56], anti-epidermal growth factor receptor antibodies in lung cancer cells [57], gemcitabine in pancreatic cancer cells [58], and cisplatin in cervical squamous cancer cells [59].

DAPK1 also has a role in cellular antiviral immune response. Once the viral infections occur, the viruses inhibit INF-mediated signals including INF-α/β for their proliferation [60,61]. After viral infection, DAPK1 enhances the activation of IFN-mediated signals through the interaction with IFN regulatory factor 3 and 7. Moreover, IFN-β increases the expression and activation of DAPK1 through the regulation of phosphorylation levels at Ser308 [62].

Besides, DAPK1 is involved in the regulation of myofibril degeneration and myocyte apoptosis induced by chronic stimulation with β1-adrenergic receptors. The result implies that DAPK1 activation might contribute to the pathogenesis of β-adrenergic receptor-related signaling during the development of heart failure [63]. 

DAPK1 seems to have both pro- and anti-inflammatory functions. It positively contributes to production and secretion of interleukin (IL)-1β in macrophages [64], while it negatively regulates inflammation in purified human T cells [65], monocytes [27], and mouse lung tissues [66]. In human diseases, ulcerative colitis (UC) was found to be closely related to DAPK1 function through inhibition of inflammation. It was also shown that DAPK1 promoter methylation led to the decrease in DAPK1 protein expression and enhanced the severity of inflammation in UC, suggesting an anti-inflammatory role of DAPK1 in UC [67]. 

Another disease that DAPK1 may be involved in is atherosclerosis. Although DAPK1 expression was increased in atherosclerotic plaques, the detailed mechanisms are not known [68]. In addition, shear stress has been reported to regulate DAPK1 expression and activity, which promotes TNF-α-induced apoptosis in cultured bovine aortic endothelial cells (ECs) [69,70]. These reports might suggest a potential role of DAPK1 in the regulation of diseases through vascular ECs.

In contrast to DAPK1 and DAPK3, DAPK2 has not been identified as a tumor suppressor in solid tumors. Interestingly, DAPK2 was predominantly found in the hematopoietic compartment [23] and emerged as a tumor suppressor in several types of leukemia [71]. Most recently, DAPK2 has also been linked to the induction of tubulointerstitial fibrosis in mice kidneys upon chronic cisplatin exposure [72] (Figure 2B). 

DAPK3 is proposed to be a tumor suppressor, suggesting that mutations in DAPK3 could result in the loss of function. It was reported that the DAPK3 gene is frequently methylated or mutated in various types of cancer [73], resulting in loss of tumor suppression via DAPK3 in cancer. In view of this evidence, DAPK3 has been regarded as a tumor suppressor. 

On the other hand, a recent study demonstrated that DAPK3 promotes cancer cell proliferation rather than the promotion of apoptosis in many types of cancer cells. In prostate cancer cells, DAPK3 promoted proliferation [74]. Furthermore, knockdown of the DAPK3 gene prevented proliferation in colon cancer cells through the inhibition of Wnt/β-catenin signals [75]. In our previous study, it was demonstrated that knockdown of the DAPK3 gene also blocked non-small cell lung cancer (NSCLC) progression via cellular signaling [76]. It was found that DAPK3 regulates proliferation, migration, and invasion through ERK/c-Myc signaling in A549 cells. These data suggest the possibility of DAPK3 as a novel therapeutic target for many types of cancer. 

In our previous study, it was found that the expression level of DAPK3 protein was increased in the mesenteric artery from spontaneously hypertensive rats (SHR) [77]. Moreover, it was found that DAPK3 promoted reactive oxygen species (ROS)-dependent vascular inflammation and thereby mediated the development of hypertension in SHR [78]. Cho et al. demonstrated in mesenteric arteries of SHR that DAPK3 modulated calyculin A-induced contraction via increasing Ca^2+^-independent Myosin light chain kinase (MLCK) activity [79]. In our previous study, it was also demonstrated that DAPK3 mediated vascular structural remodeling via stimulating smooth muscle cell (SMC) proliferation and migration [80]. Furthermore, it was demonstrated that phosphorylation of MLC2 by DAPK3 promoted smooth muscle contraction and motility [81]. These data suggest that DAPK3 might become a pharmaceutical target for prevention of hypertensive cardiovascular diseases (Figure 2C). 

Nevertheless, the data of DAPK proteins in diseases are preliminary. Further studies are needed to clarify the expression and functional correlation of DAPK proteins with these diseases.

## 6. Cross-Talk between DAPKs and ERK Signaling

ERKs are a group of MAPKs (ERK1–8) and ERK1/2 are the first discovered members of the MAPK family. Once activated (phosphorylated), ERK1/2 moves from the cytoplasm to the nucleus, which is critical for many cellular functions, such as gene transcription, cell proliferation, and differentiation [82]. Since ERK1/2 was found to be upregulated in human tumors, inhibitors of this pathway have been used for cancer therapeutics [83]. 

**Figure 3:**
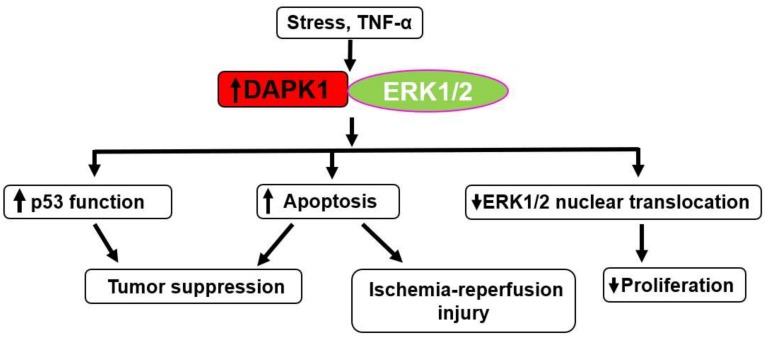
Interaction between DAPK1 and extracellular signal-regulated kinase (ERK)1/2. DAPK1-ERK1/2 interaction promotes tumor suppression and ischemia-reperfusion injury through upregulation of p53 function or propagating apoptosis. It also inhibits cell proliferation through prevention of ERK1/2 nuclear translocation. TNF = tumor necrosis factor. Up arrow indicates the increased responses, expression of protein and activity. Signal arrow indicates the promotional effects.

ERK1/2 was identified as a DAPK1-interacting protein [84] (Figure 3). DAPK1 interacts with ERK1/2 through a docking sequence within its death domain, which promotes apoptotic cell death [9,55]. On the other hand, a recent study reported that phosphorylation of DAPK1 at Ser735 by ERK1/2 leads to apoptosis in human fibroblasts [84]. This mutual regulation between DAPK1 and ERK1/2 constitutes a positive feedback circuit that ultimately enhances the apoptotic activity of DAPK1.

Mechanistically, it was shown that DAPK1 prevented activation of ribosomal S6 kinase through inhibition of ERK1/2 nuclear translocation, indicating that DAPK1 may play an inhibitory role in the survival function of ERK1/2 signaling [85]. It was recently shown that a germline mutation in the death domain of DAPK1 (N1347S) prevented the binding of DAPK1 to ERK1/2, and attenuated TNF-α-induced apoptosis [86]. 

ERK1/2 regulates activation of the p53 pathway [87], and DAPK1 activates p53 in an oncogenic signaling pathway [28]. Therefore, ERK1/2 might mediate p53 and DAPK1 pathways to maintain p53 function as a tumor suppressor.

DAPK1-ERK1/2 signals also regulate neuronal apoptosis following ischemia-reperfusion [88]. In the report, ischemia-reperfusion led to activation of DAPK1 and ERK1/2. DAPK1 was also proved to bind ERK1/2 during reperfusion following oxygen-glucose deprivation. Prevention of DAPK1- ERK1/2 binding by knockdown of the DAPK1 gene reduced neuronal apoptosis through the promotion of nuclear translocation of ERK1/2. These results reveal the potential mechanism of the DAPK1-ERK1/2 signal in the contribution to neuronal apoptosis in response to ischemia-reperfusion. Prevention of this signal pathway might become a promising therapeutic target against stroke [88].

## 7. DAPKs as Upstream Activators of Stress-Activated Protein Kinases p38 and JNK

p38 plays roles in inflammation, cell proliferation, differentiation, survival, and cell death [89,90,91,92]. While several reports showed a tumor suppressive role for p38 [93], p38 has also been involved in tumor progression by promoting cell migration, angiogenesis, and inflammatory responses [93]. 

**Figure 4:**
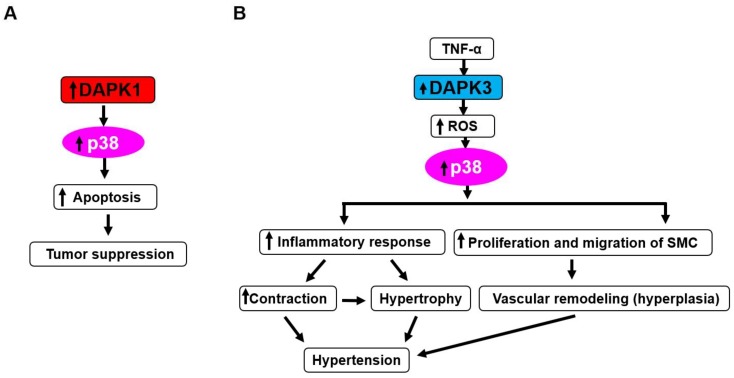
Interaction between DAPK proteins and p38. DAPK1-p38 plays a role in tumor suppression via promotion of apoptosis (**A**). DAPK3-p38 might mediate hypertension through increasing vascular contraction and hypertrophy via reactive oxygen species (ROS) generation and activation of p38-dependent inflammatory response (**B**). Besides, DAPK3 mediates vascular remodeling including hyperplasia through ROS generation, p38 activation, and promotion of proliferation and migration of SMC, which might lead to hypertension. Arrow indicates promotional effects.

Several studies provided direct evidence for the interaction of DAPK1 with p38 signaling in inflammation-associated colorectal cancer cells [94]. That study identified for the first time that p38 co-localized and interacted with DAPK1 and triggered DAPK1-mediated apoptosis in HCT116 cells. In human colorectal cancer tissues, the co-expression of DAPK1 and p38 was associated with apoptotic cell death. These results imply that a DAPK1-p38 interaction has a role in tumor suppression in colorectal cancer (Figure 4A).

In our previous study, it was clarified that DAPK3 mediates platelet-derived growth factor (PDGF)-BB-induced proliferation and migration of SMC through activation of p38/heat shock protein (HSP)27 signals, which leads to vascular structural remodeling including neointimal hyperplasia [80] (Figure 4B). Since SMC migration and vascular remodeling are important processes for the development of hypertension, these data suggest that the DAPK3/p38/HSP27 axis might be a potential pharmaceutical target for the prevention of hypertensive cardiovascular diseases [80]. These data seem to be opposite to the previous reports demonstrating that DAPK3 promoted tumor cell death [73]. This might be because DAPK3 has different functions depending on the types of cells.

JNK pathways are activated by various stress stimuli, such as heat shock, osmotic shock, ultraviolet irradiation, and cytokines [90,91,92], which mediate various functions by acting on downstream targets that include transcription factors, such as Elk1, p53, AP1, and ATF2, as well as the anti-apoptotic protein Bcl-2 [93].

**Figure 5:**
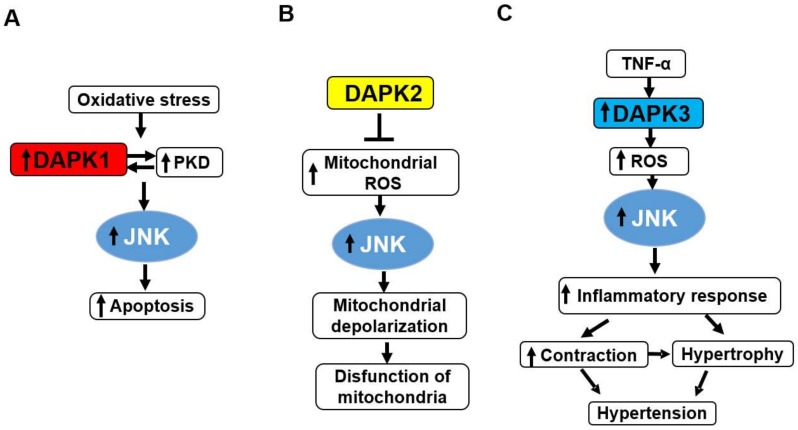
Interaction between DAPK proteins and c-Jun N-terminal kinase (JNK). DAPK1-protein kinase D (PKD) interaction promotes oxidative stress-mediated apoptosis (**A**). DAPK2 regulates the level of mitochondrial ROS through activation of JNK, which might maintain the mitochondrial functions (**B**). DAPK3 might mediate hypertension through propagating vascular contraction and hypertrophy via ROS generation and activation of JNK-dependent inflammatory response (**C**). Arrow indicates promotional effects. T bar indicates the suppressive effects.

JNK is a key node of the cell death network activated under oxidative stress [95,96,97], and DAPK1 plays a central role in oxidative stress-induced JNK signaling. It was also shown that DAPK1 mediates oxidative stress-induced JNK phosphorylation through the activation of protein kinase D, which mediates cell death [98] (Figure 5A).

In contrast, DAPK2 inhibits JNK activation through the prevention of oxidative stress in osteosarcoma and lung cancer cells [99] (Figure 5B). In the report, knockdown of the DAPK2 gene promoted mitochondrial membrane depolarization through increasing levels of mitochondrial reactive oxygen species (ROS) and phosphorylation of JNK. These data imply that DAPK2 is vital to maintaining mitochondrial integrity and cellular metabolism in cancer cells.

In our previous study, it was demonstrated that DAPK3 mediated TNF-α-induced activation of JNK, p38, and Akt through a generation of ROS [78] (Figure 4B,5C). In the report, the role of DAPK3 in vascular inflammatory responses and development of hypertension was investigated. As a result, it was found that the inhibition of DAPK3 prevented TNF-α-induced vascular cell adhesion molecule (VCAM)-1 expression and activation of JNK, p38, and Akt, as well as ROS production in vascular SMCs. It was also demonstrated that the inhibition of DAPK3 prevented TNF-α-induced expression of VCAM-1, e-selectin, and cyclooxygenase-2, as well as ROS production, in vascular endothelial cells. Since DAPK inhibitor blocked the development of hypertension in SHR and vascular inflammation, it was suggested that DAPK3 regulates hypertensive disease through vascular inflammation.

## 8. DAPKs are Therapeutic Targets of Small Molecule Inhibitors

Discovery of DAPK inhibitors has been performed using previously described experimental approaches [100,101]. Several small molecules and plant-derived compounds have been developed as inhibitors of the DAPK family kinases (Table 1). Some are selective in their action, others are not, and only a few of them have been tested in cells. It is generally considered that inhibition of DAPK proteins intercepts cell death and prevents further damage of ischemic regions in cerebral infarction and other ischemic diseases. 

Alkylated 3-amino-6-phenylpyridazine inhibited DAPK1 catalytic activity in cultured neuron cells and mouse models [41]. Furthermore, the single intraperitoneal injection of this compound reduced brain injury in an animal model when administered 6 h after the insult (hypoxia-ischemia induced injury) [41].

Another compound, (4*Z*)-4-(3-pyridylmethylene)-2-styryl-oxazol-5-one, was known to inhibit DAPK1 activity effectively and selectively (IC_50_ = 69 nM) [26,53,102]. This drug regulated DAPK1 activity-dependent neurite outgrowth and microtubule polymerization by affecting tau function [53].

The role of DAPK activity in the histone deacetylase inhibitor (LBH589)-induced apoptosis in HCT116 wildtype colon cancer cells was clarified by using a selective DAPK1 inhibitor, 2-phenyl-4-(pyridin-3-ylmethylidene)-4,5-dihydro-1,3-oxazol-5-one, known as TC-DAPK6, for inhibition of DAPK1 catalytic activity [26,103]. It was found that pre-treatment with TC-DAPK6 did not influence the LBH589-induced caspase 3-mediated cell death [103]. TC-DAPK6 acted as a novel DAPK1 and DAPK3 inhibitor (IC_50_ = 69 and 225 nM, respectively), which makes it the most potent DAPK1 inhibitor discovered to date [104].

Eighteen analogs exhibiting anti-proliferative and pro-apoptotic properties on an acute T cell leukemia cell line were obtained [105]. These analogs have a benzyloxy group at the C6 position and 9-tert-butyl-6-(benzyloxy)-8-phenyl-9*H*-purine (6d). 

Several other selective inhibitors of DAPK have also been investigated [26]. The non-selective inhibitors targeting multiple protein kinases make it challenging to dissect precisely the role of DAPK1 on certain biological processes. Among them, growing evidence of the role in DAPK1 in cancer processes are appearing [106]. Therefore, the use of selective inhibitors might shed light on the exact participation of DAPK1 in tumor development.

Besides, natural DAPK inhibitors have been developed. The chloroform fraction of *Laurus nobilis*, a DAPK1 non-selective inhibitor of plant origin, significantly improved the ischemic neuronal death through keeping DAPK1 in an inactivate form after oxygen and glucose deprivation in human SH-SY5Y neuroblastoma cell lines and organotypic hippocampal slice tissue. Moreover, it also reduced the infarct size and neurological deficit of middle cerebral artery occlusion in vivo [107].

Recently, a dual Pim/DAPK3 inhibitor (HS56) was synthesized using crystal structure-guided medicinal chemistry techniques [108]. HS56 reduced Pim kinase-induced myosin phosphorylation and the contractility of vascular smooth muscle in spontaneously hypertensive RenTG mice, suggesting a novel multi-target strategy for hypertensive diseases [108]. In the same report, selective DAPK3 inhibitors (HS94 and HS148) were also developed [108].

While specific inhibitors for DAPK2 remain to be developed, DAPK1 and DAPK3 inhibitors have been tested for DAPK2 activity. Since the catalytic domain of the three kinases shares high-sequence homology, the most potent derivative showed comparable IC_50_ value to that of DAPK1 and DAPK3 [109].

The potency of most DAPK inhibitors in in vitro kinase assays toward DAPK proteins is not very high. Improvement of the structures of these compounds is needed to gain more potent inhibitors and to perform clinical trials. Although there are only a few detailed studies focusing on DAPK proteins as a drug target, it would be helpful to use selective inhibitors against unmet medical needs.

## 9. Conclusions

Several recent studies have unraveled pathologically relevant mechanisms involving DAPK-MAPK cross-talks in neuronal diseases, cancer, and cardiovascular diseases. With the availability of more specific and effective DAPK inhibitors, the interface between DAPK and MAPKs might become promising targets in the treatment of various diseases.

## Figures and Tables

**Table 1:** Information on DAPK inhibitors.

Name	Structure	Types of Inhibitors	IC_50_
Alkylated 3-amino-6-phenylpyridazine	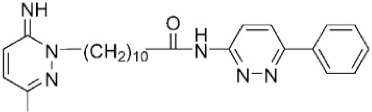	ATP-competitive	13, 22 mM for DAPK1
(4Z)-4-(3-pyridylmethylene)-2-styryl-oxazol-5-one	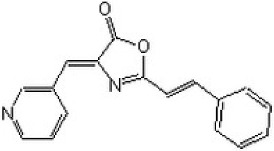	ATP-competitive	69 and 225 nM against DAPK1 and DAPK3, respectively
(4Z)-2-[(E)-2-Phenylethenyl)-4-(3-pyridinylmethylene)-5(4H)-oxazolone (TC-DAPK6)	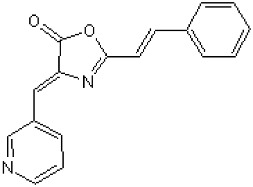	ATP-competitive	69 and 225 nM against DAPK1 and DAPK3, respectively
6-benzyloxy-9-tert-butyl-8-phenyl-9H-purine (6d)	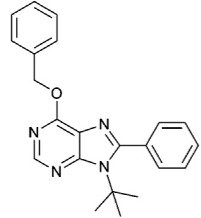	ATP-competitive	2.5 mM for DAPK1
HS56	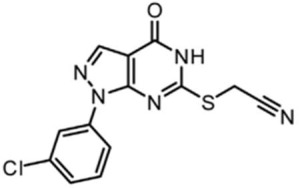	ATP-competitive	1 mM for DAPK3

Adenosine triphosphate (ATP).
