# Correction: Elbadawy, M., et al. Novel Functions of Death-Associated Protein Kinases through Mitogen-Activated Protein Kinase-Related Signals. *Int. J. Mol. Sci.* 2018, *19*, 3031

**DOI:** 10.3390/ijms21124560

**Published:** 2020-06-26

**Authors:** Mohamed Elbadawy, Tatsuya Usui, Hideyuki Yamawaki, Kazuaki Sasaki

**Affiliations:** 1 Laboratory of Veterinary Pharmacology, Department of Veterinary Medicine, Faculty of Agriculture, Tokyo University of Agriculture and Technology, 3-5-8 Saiwai-cho, Fuchu, Tokyo 183-8509, Japan; Mohamed.elbadawy@fvtm.bu.edu.eg (M.E.); skazuaki@cc.tuat.ac.jp (K.S.); 2 Department of Pharmacology, Faculty of Veterinary Medicine, Benha University, 13736, Moshtohor, Elqaliobiya, Toukh 13736, Egypt; 3 Laboratory of Veterinary Pharmacology, School of Veterinary Medicine, Kitasato University, Towada, Aomori 034-8628, Japan; yamawaki@vmas.kitasato-u.ac.jp

The authors wish to make the following corrections to this paper [1] published in *Int. J. Mol. Sci*. 2018, 19(10), 3031:

We have found that Sections 2 and 4 are the same in our paper [1]. The repeated Section 4 should be deleted, and the subsequent Section numbers need to be adjusted accordingly. The corrected Section order should be:

1. Introduction: DAPKs, MAPKs

2. Cellular Functions of DAPK Family Proteins

3. Regulation of DAPK Family Proteins

4. DAPKs in Disease

5. Cross-Talk between DAPKs and ERK Signaling

6. DAPKs as Upstream Activators of Stress-Activated Protein Kinases p38 and JNK

7. DAPKs are Therapeutic Targets of Small Molecule Inhibitors

8. Conclusions

These changes have no material impact on the conclusions of our paper. The authors would like to apologize for any inconvenience caused to the readers by these changes.
